# Efficacy and safety of left atrial appendage closure procedure in patients with non-valvular atrial fibrillation with contraindication and/or failure for oral anticoagulants: A systematic review and meta-analysis

**DOI:** 10.1016/j.clinsp.2024.100465

**Published:** 2024-08-30

**Authors:** Ricardo dos Santos Simões, Aline Frossard Ribeiro Bortoluzzi, Janaina Cardoso Nunes Marinho, Julia Simões Corrêa Galendi, Wanderley Marques Bernardo

**Affiliations:** Núcleo de Avaliação de Tecnologias em Saúde da Unimed do Brasil, Unimed do Brasil, São Paulo, SP, Brazil

**Keywords:** Atrial fibrillation, Left atrial appendage closure, Systematic review, Meta-analysis

## Abstract

**Background:**

With the aim of reducing the risk of Cerebrovascular Accident (CVA) in patients with Non-Valvular Atrial Fibrillation (NVAF), Left Atrial Appendage Occlusion (LAAO) devices are emerging as an alternative to oral anticoagulants.

**Objective:**

To analyze the efficacy and safety of the LAAO procedure in patients with NVAF and contraindications and/or failure for oral anticoagulants.

**Method:**

The search for evidence was carried out in the electronic databases Medline and Embase till January 2024. Additional searches were conducted on Google Scholar. The clinical trials registry database was also consulted. Two blinded investigators performed the search, study selection, and data collection, and assessed quality and risk of bias using the Cochrane tool for randomized clinical trials. Meta-analyses of eligible trials were performed using RevMan 5.4.1 software. The random effects model was used for all analyses.

**Results:**

Five articles were selected, among which three were non-inferiority randomized clinical trials that analyzed the performance and safety of LAAO devices compared to the use of Vitamin K Antagonists (AVKs) or Novel Oral Anticoagulants (NOACs). No randomized clinical trials were found that analyzed populations with absolute contraindications to oral anticoagulants. Having as primary outcomes analyzed the occurrence of stroke (ischemic or hemorrhagic), cardiovascular or unexplained death and systemic embolism, the non-inferiority of the LAAO procedure compared to the use of oral anticoagulants was verified.

**Conclusions:**

For patients with an absolute contraindication to anticoagulation and/or failure to use oral anticoagulants, evidence for the use of LAAO devices is scarce.

## Highlights

• Left atrial appendage occlusion is a surgical procedure considered as an alternative for populations with contraindications or failure of anticoagulation.
• Based on the studies, it was evaluated that, among the consensual metrics (CHADS-VASC and HAS-BLED), the degree of thromboembolic risk and bleeding used was variable, not clarifying the profile of patients who would benefit from the occlusion procedure.
• Most of the studies evaluated are non-inferior, comparing the occlusion of the atrial appendage of patients on anticoagulation with vitamin K inhibitors, with no contraindication to the use of this group of drugs. There are ongoing studies, with data to be published with the use of new oral anticoagulants (direct inhibitors), to compare the safety of patients undergoing atrial appendage occlusion.
• There is also a lack of evidence related to the occlusion devices, especially regarding long-term use data, since when positioned, it can cause anatomical and physiological changes. Also, there is more than one model of the device with differences between them, suggesting the need for comparative studies.

## Introduction

Atrial Fibrillation (AF) is the most common sustained cardiac arrhythmia and is associated with a high thromboembolic risk with increased and increased risk of all-cause mortality.^1^ AF is also associated with a 1.5 fold risk of cognitive impairment or dementia and a 2.33 fold risk of stroke.^2^ A systematic review including 38 studies showed that the association between atrial fibrillation and stroke is consistent, irrespective of baseline demographics or clinical characteristics.^2^ The CHA2DS2-VASc Score is the most commonly utilized method to predict thromboembolic risk in atrial fibrillation.^2^

The use of Oral Anticoagulants (OAC) effectively reduces the risk of thrombosis. OAC is recommended in patients diagnosed with AF who have an estimated annual risk of stroke or thromboembolic events equal to or higher than 2%.^3^ Although historically warfarin was the first choice of OAC, the narrow therapeutic window based on frequent monitoring and dietary restrictions limits the adherence of patients. In the Brazilian usual care context, patients in the use of warfarin were 55% of the time in the therapeutic range. Direct oral anticoagulants are non-inferior to warfarin regarding the prevention of stroke and do not require monitoring, but their use is limited due to affordability.^3^

Moreover, challenging clinical scenarios are often encountered regarding the management of stroke risk in patients with nonvalvular AF who are at high bleeding risk. According to a patient-level meta-analysis, the hazard of major bleeding in patients treated with direct oral anticoagulants is 5.05% while the hazard is slightly higher (5.95%) in patients using warfarin.^4^

The development of Left Atrial Appendage Obstruction (LAAO) devices has provided an alternative non-drug therapy in appropriately selected patients with nonvalvular AF to reduce the risk of stroke and avoid anticoagulation-related bleeding complications. The left atrial appendix is the main site of thrombus formation as a precursor to embolic stroke in patients with nonvalvular AF.^5^

This review aims to evaluate the available evidence on the efficacy and safety of the LAAO procedure in patients with NVAF and contraindications and/or failure for oral anticoagulants.

## Methodology

The authors conducted a systematic review and meta-analysis in accordance with the Cochrane Collaboration guidelines for systematic reviews of interventions,^6^^,^^7^ and reported it according to the revised Preferred Reporting Items for Systematic Reviews and Meta-Analysis (PRISMA) checklist.^6^

### Electronic databases consulted

A comprehensive and systematic search of the databases Medline/PubMed and Embase was carried out. Additional searches were conducted on Google Scholar. The ClinicalTrials.Gov registry database was also consulted. Manual searches were carried out in the reference lists of selected articles. The searches were completed in January 2024.

### Eligibility criteria

Were included studies that analyzed mechanical closure procedures of the left atrial appendage in patients with nonvalvular AF. There was no restriction on the language or date of publication. As inclusion criteria, only prospective randomized or non-randomized clinical trials whose full texts could be retrieved were selected.

### Search strategies and selection process

The search strategy was employed utilizing Boolean operators and PICO (Patient, Intervention, Control and Outcomes) criteria. Two researchers (J.C.N.M. and R.S.S.) with experience in preparing systematic reviews participated in the search for titles and abstracts independently for eligibility. Full-text articles were obtained for all potentially relevant studies. Disagreements regarding study eligibility were resolved by discussion and consensus with another investigator (A.F.R.B.). A complete description of the search strategies and Mesh terms used are found in Table 1.

**Table 1:** Databases, search strategies and MeSH terms used.

Medline/PubMed
#1: (Atrial Appendage* OR Atrium Appendage OR Appendage, Atrium OR Appendages, Atrium OR Atrium Appendages OR Auricular Appendage OR Appendage, Auricular OR Appendages, Auricular OR Auricular Appendages OR Heart Atrium Appendage OR Appendage, Heart Atrium OR Appendages, Heart Atrium OR Atrium Appendage, Heart OR Heart Atrium Appendages) n=10.683
#2: (Atrial Fibrillation* OR Persistent Atrial Fibrillation OR Atrial Fibrillation, Persistent OR Atrial Fibrillations, Persistent OR Fibrillation, Persistent Atrial OR Fibrillations, Persistent Atrial OR Fibrillation, Atrial OR Fibrillations, Atrial OR Auricular Fibrillation OR Paroxysmal Atrial Fibrillation OR Atrial Fibrillation, Paroxysmal OR Atrial Fibrillations, Paroxysmal OR Fibrillation, Paroxysmal Atrial OR Fibrillations, Paroxysmal Atrial OR Paroxysmal Atrial Fibrillations) n=110.878
#3: (Cardiac Surgical Procedures* OR Surgical Procedures, Heart OR Cardiac Surgical Procedure OR Heart Surgical Procedures OR Procedure, Heart Surgical OR Procedures, Heart Surgical OR Surgical Procedure, Heart OR Heart Surgical Procedure) n=299.347
#4: Random* n=1.724.478
#1 AND #2 n=6.714
#1 AND #2 AND #3 n=1.318
#1 AND #2 AND #3 AND #4 n=142
#1 AND #2 AND #3 n=1.318 AND ((clinical[Title/Abstract] AND trial[Title/Abstract]) OR clinical trials as topic[MeSH Terms] OR clinical trial[Publication Type] OR random*[Title/Abstract] OR random allocation[MeSH Terms] OR therapeutic use[MeSH Subheading]) n=609
**Embase**
'heart atrium appendage'/exp AND 'atrial fibrillation'/exp AND 'heart surgery'/exp AND 'controlled study'/de n=459

### Data extraction

Researchers independently extracted data from eligible studies. For each study, information regarding the first author's name and year of publication, type of study, patient characteristics, intervention, comparison, outcomes, results, and follow-up time were recorded (Table 2). Efficacy outcomes were all-cause mortality perioperative and at follow-up, cardiovascular mortality, stroke, major and minor bleeding events, transient ischemic attack and thromboembolic events.

**Table 2:** Characteristics of selected studies.

Author/Year	Study type	Patients	Intervention	Comparison	Outcomes	Results	Follow-up (months)
Holmes et al., 2009 (PROTECT AF study)	RCT, multicentric non-inferiority	*n* = 707 patients ≥ 18-years, persistent or paroxysmal NVAF, CHADS2 risk score ≥ 1 with at least one of the following characteristics: previous stroke or TIA, DM, CHF	Percutaneous LAAO device (n=463 )	Warfarin (*n* = 244)	Primary outcomes: ischemic stroke, cardiovascular or unexplained death, systemic embolism. The primary composite endpoint for safety consisted of events related to excessive bleeding or procedure-related complications	**Efficacy:** With the follow-up of 1,065 patients/year, the occurrence rate of primary outcomes was 3 per 100 patients/year (95% CI 1.9‒4.5) and 4.9 per 100 patients/year (95% CI 2.8‒7.1) for the intervention and control groups respectively	18
						**Adverse events:** Occurred more frequently in the intervention group (RR = 1.69 with 95% CI 1.01‒3.19) with the most frequent being severe pericardial effusion, which occurred in *n* = 22 patients. In the two-year follow-up after randomization, the cumulative rate of adverse events was 10.2% (95% CI 7.4‒13) for the intervention group vs. 6.8% (95% CI 3.0‒10.6) for the control group	
Holmes et al., 2014 (PREVAIL study)	RCT, multicentric non-inferiority	*n* = 407 patients with a mean age of 74-years and CHADS2 ≥ 2	Percutaneous LAAO device (*n* = 269)	Warfarin (*n* = 138)	Primary outcomes: stroke, systemic embolism and cardiovascular or unexplained death	**Efficacy:** The rate of stroke and death in the 18^th^ month of follow-up was 0.064 in the intervention group and 0.063 in the warfarin group. The calculated rate ratio was 1.07 with 95% CI 0.57‒1.89. This, however, did not meet the predefined non-inferiority criterion, as the upper limit (1.89) crosses the predefined non-inferiority margin of 1.75. The percentage per patient/year who had a stroke in the intervention group was 2.3% and in the control group 0.7%	12
Reddy et al., 2014	RCT, multicentric non-inferiority	*n* = 707 patients ≥ 18-years, persistent or paroxysmal NVAF, CHADS2 risk score ≥ 1 with at least one of the following characteristics: previous stroke or TIA, DM	Percutaneous LAAO device (*n* = 463)	Warfarin (*n* = 244)	Primary outcomes: ischemic stroke, cardiovascular or unexplained death, systemic embolism. The primary composite endpoint for safety consisted of events related to excessive bleeding or procedure-related complications	**Efficacy:** With a mean follow-up of 3.8±1.7 years (2,621 patients/year), there were 39 events among 463 patients (8.4%) in the intervention group (2.3 events per 100 patient-years) in compared to 34 events among 244 patients (13.9%) in the control group (3.8 events per 100 patient-years). Patients in the intervention group demonstrated lower rates of cardiovascular mortality (1 event per 100 patient-years for the device group [*n* = 17/463] vs. 2.4 events per 100 patient-years for warfarin [*n* = 22/244] and all-cause mortality (3.2 events per 100 patient-years for the device group [*n* = 57/466] vs. 4.8 events per 100 patient-years for warfarin [*n* = 44/244]	60
Osmancik et al., 2020 (PRAGUE-17 study NCT02426944)	RCT, multicentric non-inferiority	*n* = 415 patients with a mean age of 73.3-years and a history of bleeding that required intervention or hospitalization, history of a cardioembolic event while using an anticoagulant agent; or moderate to high risk profile, defined as CHA2DS2-VASc ≥ 3 + HAS-BLED ≥ 2	Percutaneous LAAO device (*n* = 213)	NOAC (*n* = 202)	Primary outcomes: ischemic stroke, hemorrhagic stroke or TIA, embolism, clinically significant bleeding, cardiovascular death, bleeding, and procedure/device-related complications	**Efficacy:** LAAO was successful in *n* = 181/201 patients. In the NOAC group, apixaban was used more frequently (*n* = 192/201). The annual primary outcome rates were 10.99% with OAAE and 13.42% with NOAC. There was no difference between the groups for the components of the outcome consisting of ischemic stroke, hemorrhagic stroke, TIA, clinically significant bleeding and cardiovascular death	19
						**Adverse events:** LAAO device-related complications occurred in *n* = 9 patients	
Reddy et al., 2013 (estudo ASAP NCT00851578)	CT, multicentric	*n* = 150 patients with a mean age of 72.5 ± 7.4 years and NVAF and CHADS2 risk score ≥ 1 ineligible for the use of oral anticoagulants	Percutaneous LAAO device	‒	Primary outcomes: ischemic stroke, hemorrhagic stroke, systemic embolism, and death cardiovascular/ unexplained	**Efficacy:** ischemic stroke, hemorrhagic stroke or systemic embolism occurred in *n* = 4 patients	14
						**Adverse events:** Serious safety events related to the procedure or device occurred in 8.7% of patients (*n* = 13/150)	

RCT, Randomized Clinical Trial; CT, Clinical trial; TIA, Transient Ischemic Attack; CHF, Congestive Heart Failure; DM, Diabetes Mellitus; LAAO, Left Atrial Appendage Occlusion; NOAC, New Oral Anticoagulants; CHADS2 and CHA2DS2-VASc, Stroke risk stratification scales in patients with atrial fibrillation or atrial fibrillation; HAS-BLED, One-year risk estimation scale for the occurrence of major Bleeding in patients with atrial fibrillation anticoagulated with vitamin K antagonists.

### Risk of bias and quality of evidence

The revised Cochrane Risk of Bias tool (RoB 2.0) was used to assess the risk of bias in randomized clinical trials.^8^ The RoB 2.0 is set in five domains: (1) ‘Risk of bias arising from the randomization process’, (2) ‘Risk of bias due to deviations from the intended interventions’, (3) ‘Missing outcome data’, (4) ‘Risk of bias in the measurement of the outcome’ and (5) ‘Risk of bias in the selection of the reported result’. Judgment for each domain can be assessed as “Low” or “High” risk of bias or can express “Some concerns”.^8^ Two independent reviewers investigated the risk of bias for each randomized clinical trial, and discrepancies were resolved by a third review author (W.M.B.). The evaluation of the risk of bias was done considering the main study publication, its supplementary material, and when available the published protocol and the register data from ClinicalTrials.gov. The quality of the evidence was rated as high, moderate, low or very low, according to the GRADE guidelines.^9^

### Data analysis

The meta-analysis was performed using the RevMan 5.4.1 software (Cochrane Collaboration, London, United Kingdom). Forest plot graphs were used to display the pooled estimates. A random effect model was utilized to evaluate data. The data were chiefly binary, using risk difference to evaluate effects and calculate the 95% Confidence Interval (95% CI). Heterogeneity between studies was assessed using the I² statistic. I^2^ values between 25% and 49% indicated low heterogeneity, between 50% and 75% indicated moderate heterogeneity, and an I^2^ value of 75% or above indicated high heterogeneity.

## Results

### Studies characteristics

**Fig. 1:**
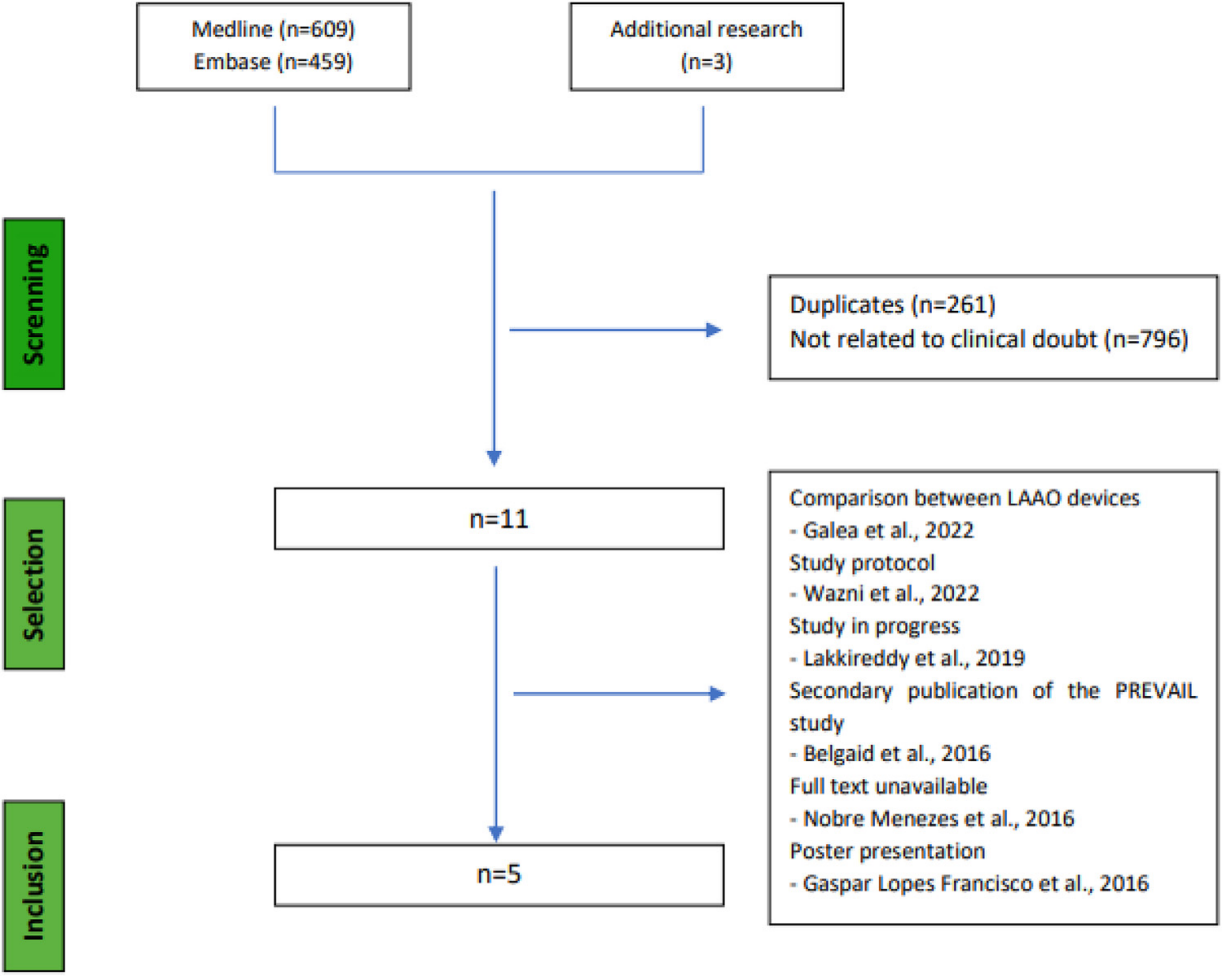
Study selection process.

The PRISMA flowchart summarizes the study selection process covering the number of articles found in the electronic databases consulted (Fig. 1). Of 1,068 references retrieved, 11 studies were screened after excluding duplicates (*n* = 261) and studies not related to the clinical question (*n* = 796). Six studies were not included because they were study protocols of ongoing studies and one poster presentation for which it was not possible to retrieve the full text.^10^, ^11^, ^12^, ^13^, ^14^, ^15^ Additional research in the Clinical trials registry database resulted in the retrieval of three studies still in progress (NCT04226547, NCT04394546 and NCT03642509). Of these 11 studies, five studies were selected, one of which, the study carried out in 2014 by Reddy et al., presents data with a prolonged period of follow-up from the original study carried out by Holmes et al., in 2009.^16^^,^^17^

The five studies included in this analysis encompass the investigation of more than 1,600 patients diagnosed with NVAF who underwent LAAO procedures or use of AVK or NOAC. With follow-up periods ranging from 14 to 60 months, outcomes related to the safety of interventions, such as excessive bleeding or complications associated with the procedure, were analyzed (Table 2). Patients were aged 18 years or older and had a CHADS2 risk score ≥ 1.

In the study carried out by Osmancik et al. (2020) patients with a history of bleeding who required intervention or hospitalization and a history of cardioembolic events even while using anticoagulant therapy were included. The 415 patients included were randomized to LAAO or NOAC. The annual primary outcome rate (consisting of ischemic stroke, hemorrhagic stroke, transient ischemic stroke, clinically significant bleeding, and cardiovascular death) was 10.99% with LAAO and 13.42% with NOAC.^17^

No randomized clinical trials were identified that analyzed the efficacy and/or safety of the LAAO procedure in patients with contraindications and/or failure for oral anticoagulants. The only study where patients were ineligible for oral anticoagulant use was the clinical trial conducted by Reddy et al., 2013.^18^ This study included 150 patients without a prospective control group. The observed ischemic stroke rate of 1.7% per year represents 77% fewer events than expected when compared to a historical cohort.^18^

Considering the heterogeneity of the population and procedures to which patients were subjected, a meta-analysis was conducted only with the results of the pivotal PROTEC AF and PREVAIL studies,^16^^,^^19^ which were two studies were non-inferiority trials comparing LAAO with AVK.

### Risk of bias

**Fig. 2:**
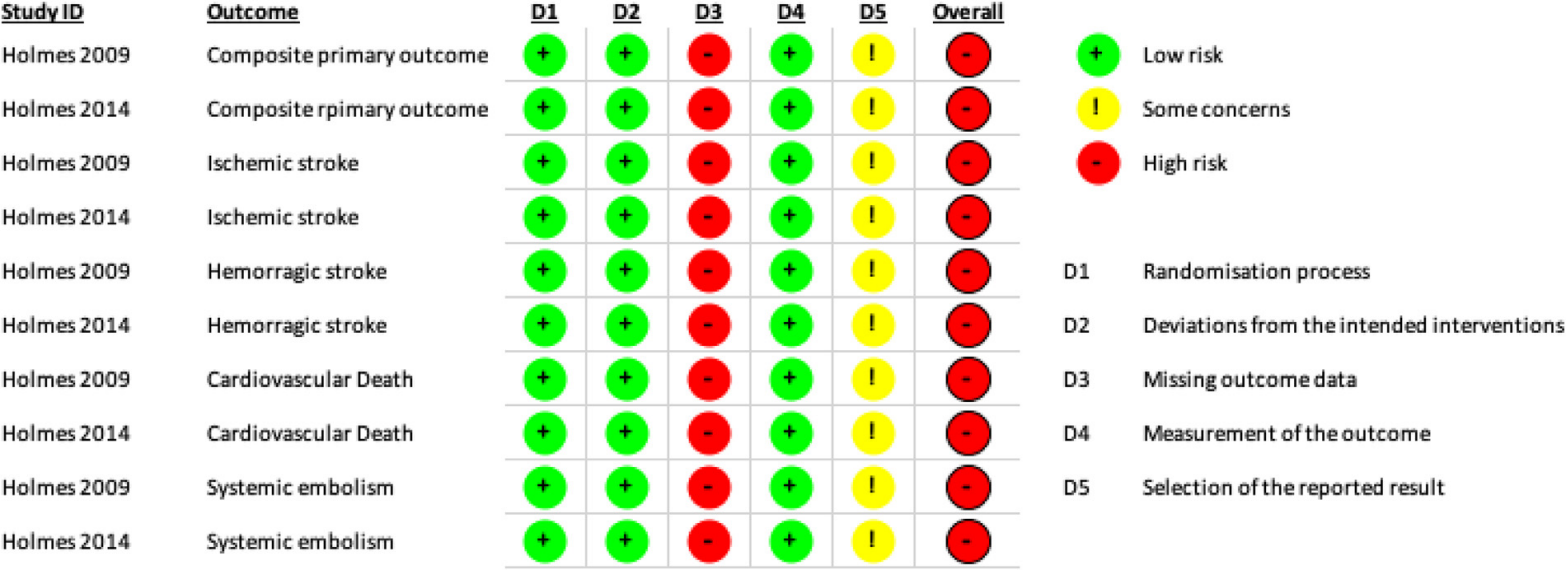
Risk of bias.

The evaluation for each domain is described in Fig. 2. The domains 1, 2 and 4 were judged to have low risk of bias. Due to the interventionist nature of the technology under study, blind allocation or blinding of participants or attending physicians involved in the analyses was not possible. Domain 3 was judged to have high risk of bias, because in both included studies there was high rates of loss of follow-up, which were unbalanced between groups. There were some concerns regarding domain 5 because neither the PROTECT AF trial^16^ nor the PREVAIL^20^ trial had their protocols published beforehand.

### Efficacy outcomes (ischemic stroke, hemorrhagic stroke, cardiovascular or unexplained death and systemic embolism)

**Fig. 3:**
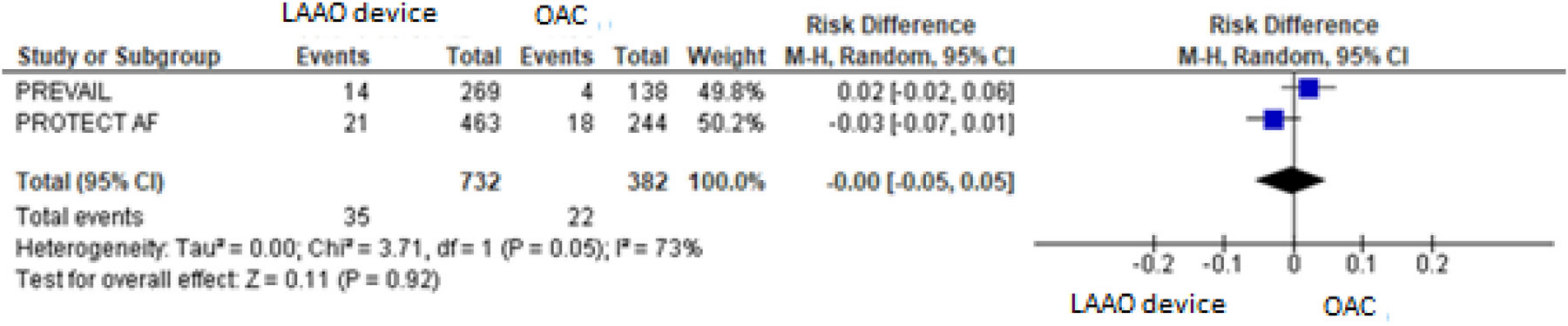
Forest plot of comparison: LAAO device and OAC, outcome: Composite primary efficacy.

**Fig. 4:**
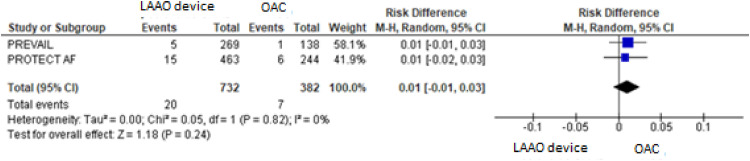
Forest plot of comparison: LAAO device and OAC, outcome: Ischemic stroke.

**Fig. 5:**
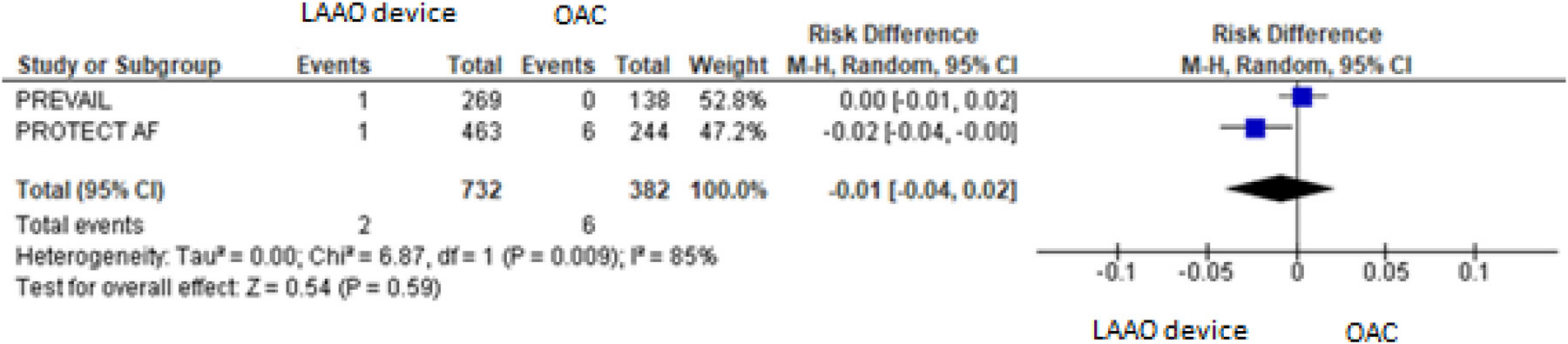
Forest plot of comparison: LAAO device and OAC, outcome: Hemorrhagic stroke.

**Fig. 6:**
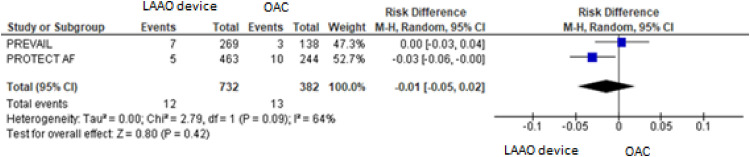
Forest plot of comparison: LAAO device and OAC, outcome: Cardiovascular or unexplained death.

**Fig. 7:**
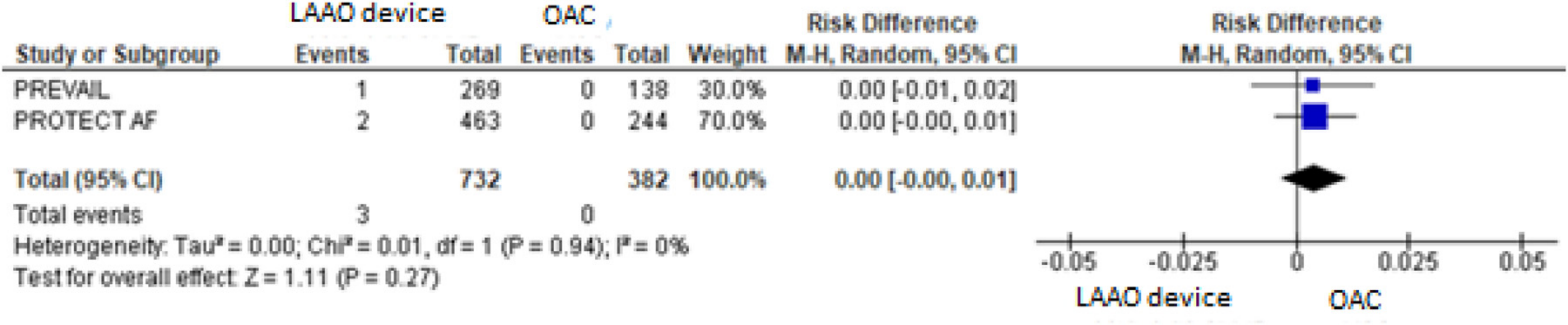
Forest plot of comparison: LAAO device and OAC, outcome: Systemic embolism.

For the composite primary efficacy outcomes (ischemic stroke, hemorrhagic stroke, cardiovascular or unexplained death, systemic embolism), there was no significant difference between the LAAO device procedure and OAC. Our pooled analysis demonstrated no significant differences in the rates of ischemic stroke and hemorrhagic stroke (RD = 0.01, 95% CI: -0.01 to +0.03, *p* = 0.24; I^2^ = 0%; RD = -0.01, 95% CI: -0.04 to +0.02, *p* = 0.59; I^2^ = 85%, respectively), cardiovascular or unexplained death (RD = -0.01, 95% CI: -0.05 to +0.02, *p* = 0.42; I^2^ = 64%) and systemic embolism (Fig. 3, Fig. 4, Fig. 5, Fig. 6, Fig. 7). The quality of the evidence is presented in Table 3.

**Table 3:** GRAADE assessment of quality of the evidence.Question: LAAO for patients with non-valvular AF and contraindications and/or failure for oral anticoagulants.

Certainty assessment							№ of patients		Effect		Certainty	Importance
№ of studies	Study design	Risk of bias	Inconsistency	Indirectness	Imprecision	Other considerations	LAAO		Relative (95% CI)	Absolute (95% CI)		
**Primary composite outcome (ischemic stroke, hemorrhagic stroke, cardiovascular or unexplained death, systemic embolism) (follow-up: 18 months)**												
2	Randomised trials	Very serious^a^	Serious^b^	Serious^c^	Serious^d^	None	35/732 (4.8%)	22/382 (5.8%)	Not estimable	**0 fewer per 1,000** (from 50 fewer to 50 more)^e^	⨁◯◯◯ Very low	CRITICAL
**Ischemic stroke (follow-up: 18 months)**												
2	Randomised trials	Very serious^a^	Not serious	Serious^c^	Serious^d^	None	20/732 (2.7%)	7/382 (1.8%)	Not estimable	**10 more per 1,000** (from 10 fewer to 30 more)^e^	⨁◯◯◯ Very low	CRITICAL
**Hemorrhagic stroke (follow-up: 18 months)**												
2	Randomised trials	Very serious^a^	Serious^f^	Serious^c^	Serious^d^	None	2/732 (0.3%)	6/382 (1.6%)	Not estimable	**10 fewer per 1,000** (from 40 fewer to 20 more)^e^	⨁◯◯◯ Very low	CRITICAL
**Cardiovascular or unexplained death (follow-up: 18 months)**												
2	Randomised trials	Very serious^a^	Serious^g^	Serious^c^	Serious^d^	None	12/732 (1.6%)	13/382 (3.4%)	Not estimable	**10 fewer per 1,000** (from 50 fewer to 20 more)^e^	⨁◯◯◯ Very low	CRITICAL
**Systemic embolism (follow-up: 18-months)**												
2	Randomised trials	Very serious^a^	Not serious	Serious^c^	Serious^d^	None	3/732 (0.4%)	0/382 (0.0%)	Not estimable	**0 fewer per 1,000** (from 0 fewer to 10 more)^e^	⨁◯◯◯ Very low	CRITICAL

CI, Confidence Interval.

Explanations:

a Due to the interventionist nature of the technology under study, blind allocation or blinding of participants or attending physicians involved in the analyzes was not possible. Domain 3 was judged to have high risk of bias, because in both included studies there was high rates of loss of follow-up, which were unbalanced between groups. There were some concerns regarding domain 5 because neither the PROTECT AF trial nor the PREVAIL trial had their protocols published beforehand.

b Heterogeneity was assessed at 73% by Higgins's test.

c No included study assessed patients with contraindication to oral anticoagulation.

d Confidence interval included the null effect.

e A risk difference meta-analysis was done.

f Heterogeneity was assessed at 85% by the Higgins test.

g Heterogeneity was assessed at 64% by the Higgins test.

## Discussion

For the composite primary efficacy outcomes (ischemic stroke, hemorrhagic stroke, cardiovascular or unexplained death, systemic embolism) and for the efficacy outcomes assessed separately, there was no significant difference between the LAAO device procedure and OAC. It is important to notice that the two trials included in the meta-analysis were non-inferiority trials.^16^^,^^20^ Non-inferiority studies are designed to evaluate whether a new treatment is substantially worse than the already established standard treatment.^21^^,^^22^ In other words, the goal is to establish that the new intervention is “non-inferior” to existing therapy in terms of efficacy by demonstrating that the evaluated intervention achieves the efficacy of the established therapy within a predetermined acceptable noninferiority margin.^21^^,^^22^ Therefore, this is a peculiar study design with null hypotheses and alternatives different from those verified for superiority studies.

The pivotal studies that evaluated the non-inferiority of LAAO devices were portrayed by two randomized clinical trials known as PROTECT AF and PREVAIL, which compared the device with AVK (warfarin) in patients with NVAF and high risk of stroke.^16^^,^^20^

In the PROTECT AF-WATCHMAN Left Atrial Appendage System for Embolic PROTECTion in Patients with Atrial Fibrillation study, *n* = 707 patients with a mean age of 71.7 ± 8.8 years and CHA2DS2-VASc score ≥ 1 (2.2 ± 1.2) were randomized in a 2:1 ratio to the LAAO procedure (*n* = 463) or use of warfarin (*n* = 244).^16^ Patients treated with the percutaneous LAAO device were maintained on anticoagulation with warfarin after the procedure and reevaluated after a period of 45 days with transesophageal echocardiography. If the occlusion of the appendage was satisfactory (prosthesis well placed, without blood flow into left atrial appendage), anticoagulation was suspended and replaced by aspirin. The primary outcome was the composite of stroke, cardiovascular or unexplained death, and systemic embolism. With a mean follow-up time of 18 months, the study showed, in the first pre-specified interim analysis, the non-inferiority of the percutaneous LAAO procedure compared to the use of oral anticoagulants (primary efficacy event rate of 3 per 100 patients/year [95% CI 1.9‒4.5] in the intervention group and 4.9 per 100 patients/year [95% CI 2.8‒7.1] in the control group presenting RR = 0.62, 95% CI 0.35‒1.25).^16^ However, the occurrence of adverse events related to the procedure was higher compared to the use of warfarin (RR = 1.69, 95% CI 1.01‒3.19).^16^ Extending the follow-up time to 3.8 years, LAAO met the non-inferiority criteria compared to the use of warfarin, for the combined outcomes of stroke, systemic embolism and cardiovascular disease.^19^

The multicenter study known as PREVAIL included 407 patients randomized to the LAAO procedure (*n* = 269) versus the use of warfarin (*n* = 138).^20^ With a design very similar to that seen in the PROTECT AF, the PREVAIL study was designed to confirm the results of that study as well as validate the safety of the device. In general, the study analyzed an older population (average age of 74 ± 7.4 years) with a higher average CHA2DS2-VASc score (2.6 ± 1.0).^20^The PREVAIL study failed to demonstrate the non-inferiority of the device versus oral anticoagulation in preventing the primary outcomes (stroke, cardiovascular or unexplained death, and systemic embolization), basically due to an occurrence of events much lower than expected in the group that had been randomized. for treatment with oral anticoagulant. However, the device was successfully implanted in 95.1% of patients with a lower rate of complications.^20^

Subsequent analysis combining data from the studies presented previously (PREVAIL and PROTECT AF) was carried out with five years of follow-up.^23^ For the PREVAIL study, the primary outcomes analyzed (stroke, systemic embolism, and cardiovascular/unexplained death) did not achieve non-inferiority. In the combined analysis of results, the primary composite outcome was similar between the groups, however, differences in the occurrence of hemorrhagic stroke, disabling/fatal stroke, cardiovascular/unexplained death, death from all causes, and bleeding after the procedure favored the intervention group, this is a group of patients who underwent LAAO.^20^ These results demonstrate that the LAAO device provides stroke prevention in NVAF comparable to that observed for warfarin, with additional reductions in major bleeding.

The multicenter study PRAGUE-17 ‒ Left Atrial Appendage Closure vs. Novel Anticoagulation Agents in Atrial Fibrillation, was a prospective randomized non-inferiority trial designed with the objective of evaluating LAAO devices compared to NOACs among patients with NVAF.^17^ Patients were eligible for the study if they had an indication for oral anticoagulation and a history of bleeding that required intervention or hospitalization. Also, among the eligibility criteria, patients with a history of a cardioembolic event during the use of oral anticoagulants and/or a combined CHA2DS2-VASc score ≥3 and HAS-BLED score ≥2 were selected. With an average follow-up time of 19.9 months, there were no differences between the groups in the occurrence of stroke, clinically significant bleeding and death related to cardiovascular disease.^17^ However, it must be observed that the duration of follow-up is relatively short, approximately 20 months, and important differences in terms of bleeding or thromboembolic events may differ at longer follow-up. Furthermore, among the devices used, a more modern generation was implanted in 2.8% of patients, which undoubtedly may have influenced the success of the procedure.^17^ However, this study is of crucial importance as it provides evidence for the use of the LAAO device in patients who have contraindication for the use of oral anticoagulants.

In the PRAGUE-17 trial, contraindication for the use of oral anticoagulant was defined as bleeding that required intervention or hospitalization. On the other hand, the American Heart Association (AHA) guideline, considers that LAAO would be reasonable for patients with long-term anticoagulation contraindicated, which includes, severe bleeding due to a nonreversible cause involving the gastrointestinal, pulmonary, or genitourinary systems; spontaneous intra-cranial/intraspinal bleeding due to a non-reversible cause and serious bleeding related to recurrent falls when causes of falls are not felt to be treatable.^3^

Other clinical trials are still ongoing with a longer follow-up period, comparing LAAO devices with NOACs in patients eligible for long-term oral anticoagulation. The CATALYST trial (NCT04226547), actively recruited, is a prospective randomized multicenter non-inferiority clinical trial. Subjects (*n* = 2,650) will be randomized to treatment with LAAO device or NOAC and will aim to analyze the safety and efficacy of the device compared to oral anticoagulant therapy in patients with NVAF and increased risk of ischemic stroke (CHA2DS2 score-VASc ≥ 3). This study is expected to be completed by 2024. The CHAMPION-AF study (NCT04394546), also in the recruitment phase, is a multicenter randomized clinical trial comparing the LAAO device with anticoagulant therapy using NOAC in a population of patients with CHA2DS2VASc score ≥ 2. The study aims to recruit *n* = 3,000 patients and is expected to be completed in the year 2027. Finally, the Occlusion-AF study (NCT03642509) is a multicenter randomized clinical trial designed to compare any LAAO device against NOAC therapy. With the primary outcome to be analyzed being the combined rate of stroke, systemic embolism, severe bleeding, and all-cause mortality, the study aims to include 750 patients and is expected to end in 2030.

Due to the concept that LAAO appears to be more appropriate for patients with contraindications to anticoagulation, the prospective non-randomized multicenter study known as ASAP-ASA Plavix Feasibility Study with Watchman Left Atrial Appendage Closure Technology was designed to evaluate the safety and efficacy of the device. In this study, 150 patients underwent device implantation and were maintained only on clopidogrel for six months after the procedure and aspirin indefinitely after the initial six months.^18^ After a mean follow-up of 14-months, the occurrence of ischemic and hemorrhagic stroke was 1.7% and 0.6%, respectively. From a multivariate analysis of patients with atrial fibrillation using aspirin and drawing a correlation based on the CHADS2 scores this ischemic stroke rate was less than that expected (7.3% per year).^18^

### Limitations

Due to the nature of the intervention with the absence of blinding of the evaluators, differences in the care of individuals undergoing the procedure can be a source of considerable bias. Regarding the meta-analysis conducted, the authors tried to homogenize both the intervention and the comparison by including only data relating to the PREVAIL and PROTEC AF studies in the analysis, however, the authors must highlight the heterogeneity of the population, both in terms of age group and thromboembolic risk.

## Conclusions

Evidence is scarce for patients with an absolute contraindication to anticoagulation. In addition, future research should clearly report the definition and the clinical criteria that constitute an absolute contra-indication to anticoagulation.

## Funding

Evidence is scarce for patients with an absolute contraindication to anticoagulation. In addition, future research should clearly report the definition and the clinical criteria that constitute an absolute contra-indication to anticoagulation.

## CRediT authorship contribution statement

**Ricardo dos Santos Simões:** Conceptualization, Data curation, Investigation, Writing – original draft. **Aline Frossard Ribeiro Bortoluzzi:** Methodology, Writing – review & editing. **Janaina Cardoso Nunes Marinho:** Data curation, Investigation. **Julia Simões Corrêa Galendi:** Writing – review & editing. **Wanderley Marques Bernardo:** Methodology, Supervision.

## Conflicts of interest

The authors declare that they are collaborators and consultants at Unimed do Brasil.
